# From milk to maturity: The potential for lactocrine programming of heifer reproduction

**DOI:** 10.3168/jdsc.2025-0842

**Published:** 2025-11-20

**Authors:** Adam D. Beard, Sabine Mann

**Affiliations:** Department of Population Medicine and Diagnostic Sciences, College of Veterinary Medicine, Cornell University, Ithaca, NY 14853

## Abstract

Standard dairy calf management sees that calves are fed a consistent liquid diet until weaning at ∼60 d of age, which is generally provided as whole milk from the bulk tank (a composite of all lactating cows) or a commercial milk replacer product. Although these diets aim to satisfy the nutritional needs of the calf, their composition is not similar to the transient profile of hormones and nonnutritive bioactive factors in the milk the dam produces at this stage. Inclusion or exclusion of hormones in milk during this time may have substantial effects on reproductive programming via lactocrine signaling. This review proposes 4 potential lactocrine factors (IGF-1, insulin, estradiol, and progesterone) relevant to current dairy calf feeding strategies and highlights known interactions within female reproductive development, wide knowledge gaps, and opportunities for further investigation.

## Highlights

• A multitude of preweaning dairy calf feeding strategies exist.
• Bulk whole milk and early maternal milk are not similar in hormone content.
• The prepubertal ovary, uterus, and brain are responsive to hormone exposure.
• The effects of early exposure to maternal hormones in milk are plausible, but unknown.
• The lactocrine contributions of milk may drive optimal heifer reproductive development.

## Graphical Abstract

**Figure:**
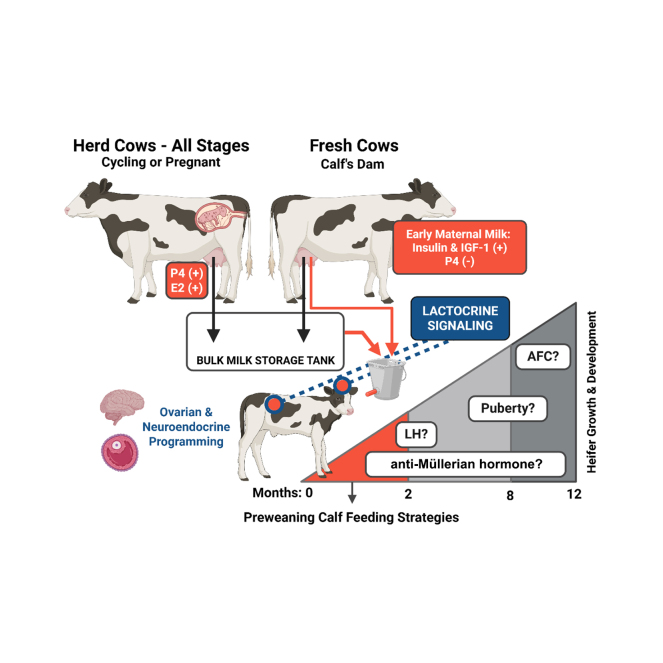
**Summary:** A dairy calf is generally fed a liquid diet for 60 days, which is often sourced from all herd cows or a commercial milk replacer product. The milk of cycling or pregnant herd cows contains greater concentrations of steroid hormones (progesterone [P4] and estradiol [E2]) compared with that of a fresh cow (the calf's dam). On the contrary, the early maternal milk (transition milk) of a cow is greater in insulin and insulin-like growth factor-1 (IGF-1) content. Each of these hormones has the potential to regulate prepubertal heifer reproductive and neuroendocrine physiology, which is consistent with the “lactocrine hypothesis.” The effects on circulating luteinizing hormone (LH) and anti-Müllerian hormone (AMH), ovarian antral follicle count (AFC), and timing and maintenance of puberty are all of interest but are not well studied in the existing literature. Leveraging the lactocrine effects of bulk milk or maternal milk consumption may drive female reproductive programming and optimize current management practices.

Replacement heifers are costly members of a dairy herd, as they require 2 or more years of investment and offer no guaranteed output (Akins, 2016). In beef cattle, heifers that are able to attain and maintain ovarian cyclicity (i.e., puberty) are younger at first breeding and have greater fertility, characterized by fewer repeat breedings (Nafziger et al., 2021). Translated into the dairy system, this would imply that heifers would be introduced to the milking herd without significant delay, ultimately mitigating costs by shortening the nonproductive phase. The role of nulliparous heifers is critical, as they are tasked with producing daughters and accelerating herd genetics through the predominant use of sex-sorted semen (Lauber et al., 2023). This suggests that robust and timely pubertal development is crucial for reproductive and productive potential, and for the success of a dairy operation as a whole. Assembly of reproductive and endocrine tissues and the programming of their function begin in utero and are supported or damaged by the dam's management, environment, and exposures (Martin et al., 2007; Yang and Fortune, 2008; Dado-Senn et al., 2021). Continuation of this relationship via maternal milk (**MM**) consumption in the first days and weeks of postnatal life is probable. Evidence predominantly from pigs proposes a “lactocrine hypothesis” in which milk-borne bioactive factors (**MbF**) are key components of postnatal uterine development in gilts (Bartol et al., 2009; Bagnell and Bartol, 2019). However, the relationship between early life feeding and reproductive outcomes has not been diligently studied in the same context in cattle. Therefore, we propose that applying the lactocrine hypothesis (i.e., effects on reproductive development mediated by the source, composition, quality, or quantity of milk provided to heifer calves during the early postnatal and preweaning period), is an area of opportunity for optimizing and supporting reproductive programming.

The term “lactocrine” was first used in 2006 to describe the stimulation of the uterine relaxin (**RLX**) receptor in neonatal gilts that experienced increased circulating RLX after being allowed to suckle from their dam in the first 48 h after birth compared with gilts fed a RLX-free milk replacer, demonstrating milk as a maternal source of RLX (Yan et al., 2006). Further investigation by feeding milk replacer (**MR**) instead of colostrum or MM generated a lactocrine-null condition in which the endometrial development of gilts was stunted at 2 and 14 d of age (Miller et al., 2013). Lactation of the tammar wallaby, in which individual mammary glands produce milk of differing composition for various aged offspring, demonstrates nature's priority for MM synchronous with postnatal age (Hendry et al., 1998). At the time of this review, and less than 20 years since the first use, a PubMed inquiry of the term “lactocrine” produces just 32 search results, of which only a fraction are research articles. Lactocrine signaling was recently implicated in a comprehensive review of female developmental programming that included the dairy calf; however, speculation was limited due to lack of research (Akbarinejad and Cushman, 2024). Our review was inspired by the multitude of dairy calf rearing strategies that are currently in practice, many of which limit MM consumption, thereby limiting the potential lactocrine impact of MbF on postnatal programming of female bovine reproductive tissues and function. Despite lack of direct evidence of the lactocrine hypothesis for reproductive programming in the bovine, we targeted our review on 4 factors relevant to current dairy calf feeding strategies. We chose these based on their presence in MM and their role in the reproductive biology and neuroendocrinology surrounding female pubertal development: (1) IGF-1, (2) insulin, (3) estradiol (**E2**), and (4) progesterone (**P4**).

Gonadotropins (LH and FSH), growth factors (insulin and IGF-1), and steroids (E2 and P4) are key players with distinct roles in bovine reproductive endocrine function. In female cattle, puberty, or first ovulation, is marked by a coordinated shift in the endocrine and ovarian follicle dynamics, which must occur in order to achieve sexual maturity, breeding eligibility, and entry into the productive herd. Notably, the pulsatile release of LH from the anterior pituitary increases in frequency near the onset of puberty. In the prepubertal phase, LH pulse frequency is low (∼2 pulses/24 h) compared with mature heifers (>20 pulses/24 h; Kurz et al., 1990; Gomez-León et al., 2020). This shift is related to E2 negative feedback on GnRH, the hormone that induces the release of LH and FSH. In mature heifers and cows, LH pulses are less frequent in the presence of high P4 concentrations. Mature (cycling) heifers with no detectable P4 are observed to have 9.6 LH pulses in a 10-h period, versus 4.1 pulses in heifers with high P4 concentrations (Gomez-Leon et al., 2023). Another key player in the ovarian and neuroendocrine physiology surrounding puberty is IGF-1 (Spicer and Echternkamp, 1995; Fortes et al., 2013; Dees et al., 2021). Early evidence from studies conducted in a rodent model show that IGF-1 stimulation of GnRH neurons advances puberty. In mice, systemic administration of IGF-1 results in earlier puberty, but not when the IGF receptor on the GnRH neurons is knocked out (DiVall et al., 2010). Insulin-like growth factor-1 also acts in the bovine ovary. Ovarian follicle formation begins in utero, and individual follicles remain dormant until activated (Yang and Fortune, 2008; Fortune et al., 2010). Primordial follicle activation (**PFA**) is stimulated by insulin, which prompts development through the preantral and antral stages of folliculogenesis (Yang et al., 2017). In contrast, IGF-1 is described to not support, and may even inhibit, PFA (Fortune et al., 2004). Early life exposure to sources high in insulin or IGF-1 could differentially alter the rate of PFA, the size of the ovarian reserve, the antral follicle count (**AFC**), and anti-Müllerian hormone (**AMH**) concentrations. These parameters may have implications for fertility and longevity in the herd (Ireland et al., 2008, 2011; Mossa and Ireland, 2019).

Driving reproductive success by decreasing preweaning calf diseases may be achieved by prioritizing greater consumption of colostrum and MM, in turn, mitigating the effects of prior disease on decreasing insemination, pregnancy, and first calving hazards (Abuelo et al., 2021a,b). Colostrum feeding protocols for replacement heifers are farm-dependent and vary due to the volume fed as a percent of birth BW, number of feedings, and storage and preparation (Shivley et al., 2018; Westhoff et al., 2023). Calves that do not have adequate transfer of passive immunity have only a ∼55% likelihood of remaining disease-free until 60 d of age (Urie et al., 2018). High incidence of health events, such as bovine respiratory disease during the prepubertal period, is associated with decreased reproductive efficiency, milk production, and longevity in the herd (Stanton et al., 2012). Therefore, we acknowledge that colostrum and early MM feeding are likely to support reproductive success independent of potential lactocrine mechanisms.

Milk can be categorized into a few types, each of which has transient hormone profiles depending on stage of lactation. Over the first few days and weeks postpartum, bovine mammary secretions are dynamic in composition and will transition (transition milk; **TM**) from colostrum to mature whole milk (**WM**). Notably, total solids (%), fat (%), and total protein (%) are decreased in WM compared with colostrum and TM, however, the opposite is true for lactose (%; Godden, 2008). Likewise, IGF-1, insulin, E2, and P4 in milk are transient over the course of lactation. Both IGF-1 and insulin signal via the IGF-1 receptor (IGF1R; Belfiore et al., 2009). These 2 hormones are present in bovine milk at high concentrations postpartum and decrease as lactation progresses (Malven et al., 1987; Collier et al., 1991; Vega et al., 1991). In colostrum, IGF-1 concentrations are 10-fold greater than in the second week of lactation (6.3 ng/mL), and continue to decrease to 3.2 ng/mL in wk 3 and 4 and 1.6 ng/mL in mo 2 and 3 of lactation (Collier et al., 1991). Concentration and yield of IGF-1 is not different between parities, but insulin concentration and yield is greater in the colostrum of primiparous versus multiparous cows (Fischer-Tlustos et al., 2025). Sex-sorted semen to produce daughters is predominantly allocated to nulliparous heifers, which would result in primiparous cows supplying insulin-rich colostrum, with the potential of greater lactocrine signaling by insulin via IGF1R, to most a farm's replacement heifer calves. We propose that increased circulating IGF-1 and insulin in the calf due to feeding MM opposed to WM in early life may alter the regulation of ovarian follicle activation and neuroendocrine signaling at the hypothalamus relevant to the attainment of puberty in dairy heifers.

Postpartum anovulation is a condition in which cows do not have ovarian cyclicity, and thus no luteal production of P4 due to the homeorhetic adaptation to prioritize milk synthesis in early lactation (Butler and Smith, 1989; Beam and Butler, 1999). The duration of anovulation is cow dependent and can be categorized into various reproductive disorders or phenotypes (Wiltbank et al., 2002; Mann et al., 2005). A study conducted in 54 postpartum Holstein cows found that 53.7% of the group had not resumed cyclicity by 40 DIM (Staples et al., 1990). Likewise, greater than 3,500 observations from Nordic Red, Holstein, and Nordic Red × Holstein cows demonstrated average resumed luteal activity at ∼39 DIM (Mäntysaari et al., 2022). In beef cows, this phenomenon can be perpetuated by a suckling calf, or the presence of a calf alone, regardless of suckling status (Hoffman et al., 1996; Williams et al., 1996). Milk P4 concentration only begins to increase with the onset of cyclicity and peaks in pregnant cows at 90 d of gestation (26.2 ng/mL), corresponding to 5.3 ng/mL in the plasma (Ginther et al., 1974). Similarly, E2 concentrations in Holstein WM are greatest in cows that are 141 to 210 d pregnant compared with the MM of nonpregnant early-lactation cows (Pape-Zambito et al., 2007). Therefore, young calves fed MM naturally consume minimal P4 and E2. In-line detection of P4 in milk is used in specialized milking systems and parlors to diagnose the reproductive status of lactating cows; thus, it is not an unknown component of the WM fed to calves (Bruinjé and Ambrose, 2019).

Herein, we explore the previously described types of milk fed to calves, and their effects on milestones within heifer reproductive development. Three independent studies published between 1997 and 2010 explore the growth, puberty, and reproductive outcomes in Israeli Holstein heifers fed with different milk feeding strategies. When comparing WM to MR feeding, 60 d of feeding WM compared with MR resulted in an earlier age at first artificial insemination (**AI**; 426 vs. 452 d), no difference in age at first conception, and a younger age at first calving (705 vs. 750 d; Moallem et al., 2010). Only 10 of the 44 heifers reached puberty by 320 d of age: 8 of 22 for WM and 2 of 22 for MR (Moallem et al., 2010). On the contrary, Shamay et al. (2005) found that WM or MR feeding until 50 d of age results in earlier puberty for WM than MR, and no effect on BW at puberty, or on age or BW at first calving. Bar-Peled et al. (1997) fed calves MR or allowed calves to suckle from their dam (MM) 3 times per day for 6 wk. Calves that were allowed to suckle were younger at first conception (394 vs. 426 d) and at first calving (669 vs. 700 d). Conception rate and BW at conception tended to be higher for MM. From these 3 studies, it appears that compared with feeding MM or WM, feeding MR may contribute to delays in age at puberty, first AI, conception, and calving, and thus a delay in entering the milking herd. However, it is unclear if MM and WM-fed calves differ. Therefore, to better understand (1) the effects of WM and MM versus MR and (2) possible lactocrine factors present in MM or WM that influence heifer reproductive development, a controlled comparison of MM and WM sourced from a bulk tank is needed, and with the inclusion of an MR-fed group. Without knowing the exact composition of the WM, MM, and MR of these studies, we may infer that the MR-fed calves have different reproductive development due to lactocrine-null or lactocrine-deficient conditions. Dried skim milk (defatted WM) used in MR is described to have lower estrogen content compared with WM, as the lipid-soluble maternal steroids are likely to be removed in large during the defatting process (Wolford and Argoudelis, 1979). However, this does not account for the possible hormonal contributions from added animal fats (tallow and lard) in MR formulation (Chiesa et al., 2016). Analytical techniques for measuring such compounds in MR are emerging, some of which report that estrogens are not detected (Wang et al., 2011).

Few studies have directly tested the programming effects of potential lactocrine factors in the prepubertal bovine female, fewer in the dairy heifer, and seemingly none at all in the milk-fed calf. A series of studies explored the effects of including E2 treatment following MR feeding on gross anatomical measurements of the female reproductive tract (Wilson et al., 2017). This was studied in combination with both a restricted (restricted diet [**RD**]: 20.9% CP, 19.8% fat; fed at 0.44 kg of MR powder/d) and enhanced (enhanced diet [**EH**]: 28.9% CP, 26.2% fat; fed at 1.08 kg of MR powder/d; Wilson et al., 2017) plane of preweaning nutrition. In the first study, 2 groups of Holstein heifers were fed RD or EH diets until 8 wk of age, then euthanized. No E2 treatment was included in study 1. Body weight was increased in EH-fed calves (81.7 vs. 52.3 kg), in addition to increased total wet tract and uterus weights. When adjusted for BW, the whole tract and uterus weights were not different. In study 2, heifers were fed according to study 1, then immediately postweaning received an E2-containing or blank silicon ear implant for 2 wk, and were then euthanized at 10 wk of age, resulting in 4 groups total (RD + E2, RD + blank, EH + E2, and EH + blank). The implants were expected to achieve 70 to 120 pg/mL of circulating E2 concentrations. As in study 1, EH resulted in increased BW, and it was further increased with E2 supplementation (EH + blank: 73.0 vs. EH + E2: 83.1 kg). The E2-stimulated boost in BW gain was not observed for RD-fed calves. In wk 10, the total and adjusted reproductive tract weights were increased in EH + blank and were further increased by the E2 implant, in EH + E2 calves only. Independent of diet, E2 increased ovarian follicle number per gram of ovarian tissue; however, ovary weight adjusted for calf BW was not altered. It is unclear if the effects of E2 on follicle number at wk 10 of age are considered desired or detrimental for development. Folliculogenesis is a dynamic process and requires repeated assessment of the same ovary and population of follicles; thus, the greater AFC in response to E2 may not be sustained long-term. Additionally, serial evaluations of prepubertal uterine size and thickness by ultrasound may prove useful (Honaramooz et al., 2004). Both E2 and P4 are expected to decrease the pulsatile release of GnRH before puberty; thus, short-term dysregulation of follicle growth may be possible, but may not be evident of changes in follicle biology or population later in life. In prepubertal beef heifers (∼320 d of age), 10 d of synthetic P4 (progestin) treatment decreased LH pulsatility; however, LH pulses increased compared with control following the removal of the implant (Anderson et al., 1996). Additionally, the progestin-treated heifers had an accelerated age to puberty (Anderson et al., 1996). From these studies, we learn that prepubertal exogenous steroid exposure alters the development of female reproductive tissues, endocrine function, and expedites age at puberty, however, we need further investigation of their effects from consumption during the preweaning phase.

From the collective results discussed in this review, we cannot define a clear relationship between the composition of milk fed in the preweaning phase, the exposure to potential lactocrine factors, and the reproductive programming and endocrine biology surrounding puberty of the female bovine. However, we do have evidence that a variety of calf rearing systems have led to changes in the development of reproductive tissues, age at puberty, first insemination, first conception, and first calving, all of which are key determinants in the timing and success of a heifer entering the productive herd. We propose that further investigation into the biological impact of these calf rearing strategies and the composition of milk or milk-based diet is needed in order to (1) best leverage their effects for supporting pubertal development and programming of reproductive tissues and function, and (2) optimize heifer reproductive management and milestones according to the herd-dependent preweaning system being used.
